# Feasibility of a surveillance programme based on gargle samples and pool testing to prevent SARS-CoV-2 outbreaks in schools

**DOI:** 10.1038/s41598-021-98849-1

**Published:** 2021-09-30

**Authors:** Catherine M. Sweeney-Reed, Doreen Wolff, Sarah Hörnschemeyer, Henriette Faßhauer, Antonia Haase, Dirk Schomburg, Jakob Niggel, Michael Kabesch, Christian Apfelbacher

**Affiliations:** 1 Neurocybernetics and Rehabilitation, Dept. of Neurology, Medical Faculty, Otto von Guericke University Magdeburg, Leipziger Str. 44, Magdeburg, Germany; 2 Center for Behavioral Brain Sciences, Otto von Guericke University Magdeburg, Magdeburg, Germany; 3 Institute of Social Medicine and Health System Research, Otto von Guericke University Magdeburg, Magdeburg, Germany; 4 Institute of Biometry und Medical Informatics (IBMI), Otto von Guericke University Magdeburg, Magdeburg, Germany; 5 University of Regensburg, Regensburg, Germany; 6 MaganaMed GmbH, Regensburg, Germany; 7 University Children’s Hospital Regensburg (KUNO), Hospital St. Hedwig of the Order of St. John, Regensburg, Germany; 8 Research and Development Campus Regensburg (WECARE), Hospital St. Hedwig of the Order of St. John and University of Regensburg, Regensburg, Germany

**Keywords:** Disease prevention, Policy and public health in microbiology, SARS-CoV-2, Viral transmission

## Abstract

School closures have a negative impact on physical and mental well-being, and education, of children and adolescents. A surveillance programme to detect asymptomatic SARS-CoV-2 infection could allow schools to remain open, while protecting the vulnerable. We assessed the feasibility of a programme employing gargle samples and pool testing of individually extracted RNA using rRT-qPCR in a primary and a secondary school in Germany, based on programme logistics and acceptance. Twice a week, five participants per class were selected to provide samples, using an algorithm weighted by a risk-based priority score to increase likelihood of case detection. The positive response rate was 54.8% (550 of 1003 pupils). Logistics evaluation revealed the rate-limiting steps: completing the regular pre-test questionnaire and handing in the samples. Acceptance questionnaire responses indicated strong support for research into developing a surveillance programme and a positive evaluation of gargle tests. Participation was voluntary. As not all pupils participated, individual reminders could lead to participant identification. School-wide implementation of the programme for infection monitoring purposes would enable reminders to be given to all school pupils to address these steps, without compromising participant anonymity. Such a programme would provide a feasible means to monitor asymptomatic respiratory tract infection in schools.

## Introduction

A pandemic was declared by the World Health Organisation in March, 2020 due to spread of a new form of coronavirus, which can result in severe acute respiratory syndrome (SARS-CoV-2 virus)^1^. School closures ensued in many countries worldwide as a part of a wider lockdown strategy to limit viral spread through minimising social contacts. Despite initially reduced infection incidence, a third wave of new infections developed in Germany in Autumn 2020, and schools were closed again. Many months will be required, before all populations have been offered vaccination, and new virus mutations, with higher infectivity, remain a potential cause for concern. Hygiene measures applied to limit infection spread include regular disinfection, wearing of protective masks covering the nose and mouth, alertness to symptom development, regular room ventilation, and social distancing^2,3^. Despite implementation of these measures, however, schooling on alternating days, as well as school closures, remain among the measures applied to bring infection rates under control.

School closures have a negative impact not only on education but also on the physical and mental well-being of children and young people, however^4–10^, with children in socially disadvantaged circumstances particularly affected^11^. The development of a strategy with which schools can safely remain open, while protecting the vulnerable, is therefore paramount both during the current pandemic, as well as potentially in future circumstances in which a highly transmissible respiratory virus with high morbidity and mortality enters the population.

Regular testing of entire populations was proposed early in the pandemic as a potential strategy for limiting viral transmission^12^. Surveillance, with targeted contact-tracing and quarantine measures, has moreover been recommended for schools in particular^13–15^. Modelling of the temporal development of viral load in SARS-CoV-2 cases indicates that most SARS-CoV-2 transmission occurs in the pre-symptomatic stage^16^. Furthermore, children in the acute phase of infection frequently have an asymptomatic course^17^. These findings suggest that regular testing of asymptomatic pupils would be required to prevent infection outbreaks.

**Figure 1﻿:**
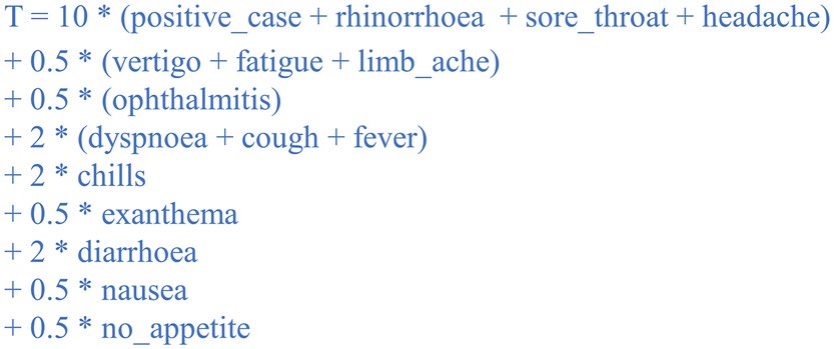
Algorithm used to increase the likelihood of including positive cases in pools for testing by computing a priority score based on the variables collected in the Initial (Q1) and Pre-test Questionnaires (Q2). Each variable was set to 1 if true and 0 if false. The threshold (T) was set to reflect local infection incidence levels, such that no more than 5 pupils were selected for each test round.

We developed a protocol for a surveillance programme based on gargle samples and pool testing and evaluated its feasibility in two model schools, a primary and a secondary school in Magdeburg, Germany (Study of Coronavirus Outbreak Prevention in Magdeburg Schools: STudie zur Ausbruchsvermeidung von CoronA an MAgdeburger Schulen [STACAMA]). A detailed Study Protocol is available^18^. The approach is adapted from a concept developed at the University Children’s Hospital Regensburg of East Bavaria, and Hospital St. Hedwig of the Order of St. John, Regensburg, Germany, which was applied in a choir-based boarding school (Study of Coronavirus Outbreak Prevention in the Cathedral Choir School: STudie zur Ausbruchsvermeidung von CoronA bei den Domspatzen [STACADO]). We arranged pool tests, including samples from up to five pupils, twice a week for each participating class. The five participants per class were selected using a weighted algorithm to raise the probability of identifying a positive case (Fig. 1). If a new case arises in a class of 25 pupils, the chance of randomly selecting that particular individual is 20% per testing day. Given the limited access to PCR testing at the time of the study due to saturated laboratory capacities, 20% constitutes a vast improvement over the alternative of foregoing testing. Our use of a risk-based priority score was expected to increase the chances of detecting a positive case further, which is dependent on local incidence rates at the time of testing. The key difference between the current study (STACAMA) and the study in Regensburg (STACADO) is that our model schools are attended by pupils on a daily basis, whereas STACADO was performed in a boarding school. Our approach necessitated the involvement of the pupils’ families, as families were required to provide information regarding symptoms and contacts twice weekly via the study app to weight the algorithm, and pupils collected samples for testing at home.

Gargle samples were chosen as the test material for several reasons. Gargling is non-invasive and easy to perform, which is essential for regular testing of asymptomatic children. Moreover, sample collection can be carried out independently at home, avoiding potential viral spread through testing in schools and minimising costs for personnel and protective clothing^19,20^. Gargle sample testing has proven effective in diagnosing viral respiratory tract infections in children^21^ and has specifically been demonstrated to enable detection of SARS-CoV-2 infection in self-collected samples^22–24^. Nasopharyngeal swab (NPS) sampling has been considered the gold standard in SARS-CoV-2 diagnosis, and gargle sample testing has been shown to provide comparable sensitivity and specificity on polymerase chain reaction (PCR) testing^20,25^. Although slightly lower virus SARS-CoV-2-specific ribonucleic acid (RNA) concentrations were detected in gargle than NPS samples in a study including 34 patients^26^, sensitivity was found to be comparable to that of using NPS swabs in a study in which 90 of the 340 participants were asymptomatic at the time of sampling^25^. A pool testing procedure can be applied to gargle samples from several individuals for testing, offering an efficient and cost-effective approach for regular population monitoring^27,28^. A modelling study has recently shown that prevalence can be estimated based on relatively few pool tests, with identification of around twenty times as many cases through pool testing as through individual testing with comparable costs^29^.

Regular rapid antigen testing through self-administered anterior nasal swabs was introduced into German schools on a compulsory basis following school reopening after the recent lockdown. While immediate result availability is advantageous, collecting samples in schools carries a risk of viral transmission when masks are removed, and the specificity is lower than that of PCR tests^30^. Subsequent PCR-based confirmation of positive results is thus required. Indeed, several German medical societies have expressed concern over the high rates of false positive and false negative results of rapid antigen tests and called for evaluation of the logistics of surveillance programmes based on pool testing as an alternative^31^. The parallel employment of two testing approaches in the model schools, that of the current surveillance programme and the independently established compulsory programme introduced into the schools based on rapid antigen testing, enabled a direct comparison of the acceptability of the two methods among pupils and families with direct experience of both.

Key to the success of a surveillance programme in schools are the logistics and acceptability. Here we assess the logistics of our proposed programme in a step-wise approach and report evaluation of the acceptance of the programme among pupils and their families. The logistical steps include the practical implementation of the testing programme as well as rapid communication of results in accordance with stringent data protection standards. Acceptance was based on study participation rates and two acceptance questionnaires. The first acceptance questionnaire explored reasons behind the choice over participation. The second questionnaire examined preferences over test procedures and included a comparison with the acceptance of the compulsory rapid antigen testing independently introduced into the schools.

## Methods

### Study population

The study population included pupils attending a primary school (grades 1–4, pupils aged 6–10 years, 2 classes per grade, 20–24 pupils per class) and a secondary school (grades 5–12, pupils aged 10–18 years, 4 classes per grade, 22–30 pupils per class, except for the eleventh and twelfth grades, with 93 and 96 pupils respectively, in which pupils are no longer in class cohorts) in Magdeburg, Germany. A prerequisite to starting testing was a minimum of 60% participation per class, which was subsequently reduced to 50% based on interim analyses presented in the Results section. Additional classes could be included throughout the 16-week study period, on reaching the minimum participation threshold.

### Questionnaires

We employed four types of questionnaire, which we define here for clarity. Two questionnaires were completed via the study app, and the answers were used to weight the participant selection algorithm used directly before each test round to select the pupils who would provide samples. These were Questionnaire 1 (Q1), the Initial Questionnaire, which was completed on initial inclusion in the testing programme, and Questionnaire 2 (Q2), the Pre-Test Questionnaire, which was completed before each test round. The rates of their successful completion formed a part of the evaluation of the logistics of the surveillance programme. Two further questionnaires addressing the acceptance of the programme among the families (parents and/or legal guardians) of the pupils of the two schools were completed either online or in paper format. Questionnaire 3 (Q3) addressed reasons behind the choice over participation in the study. Questionnaire 4 (Q4) focused on the test method used in the surveillance programme and preferred test method choices.

### Procedure

**Figure 2:**
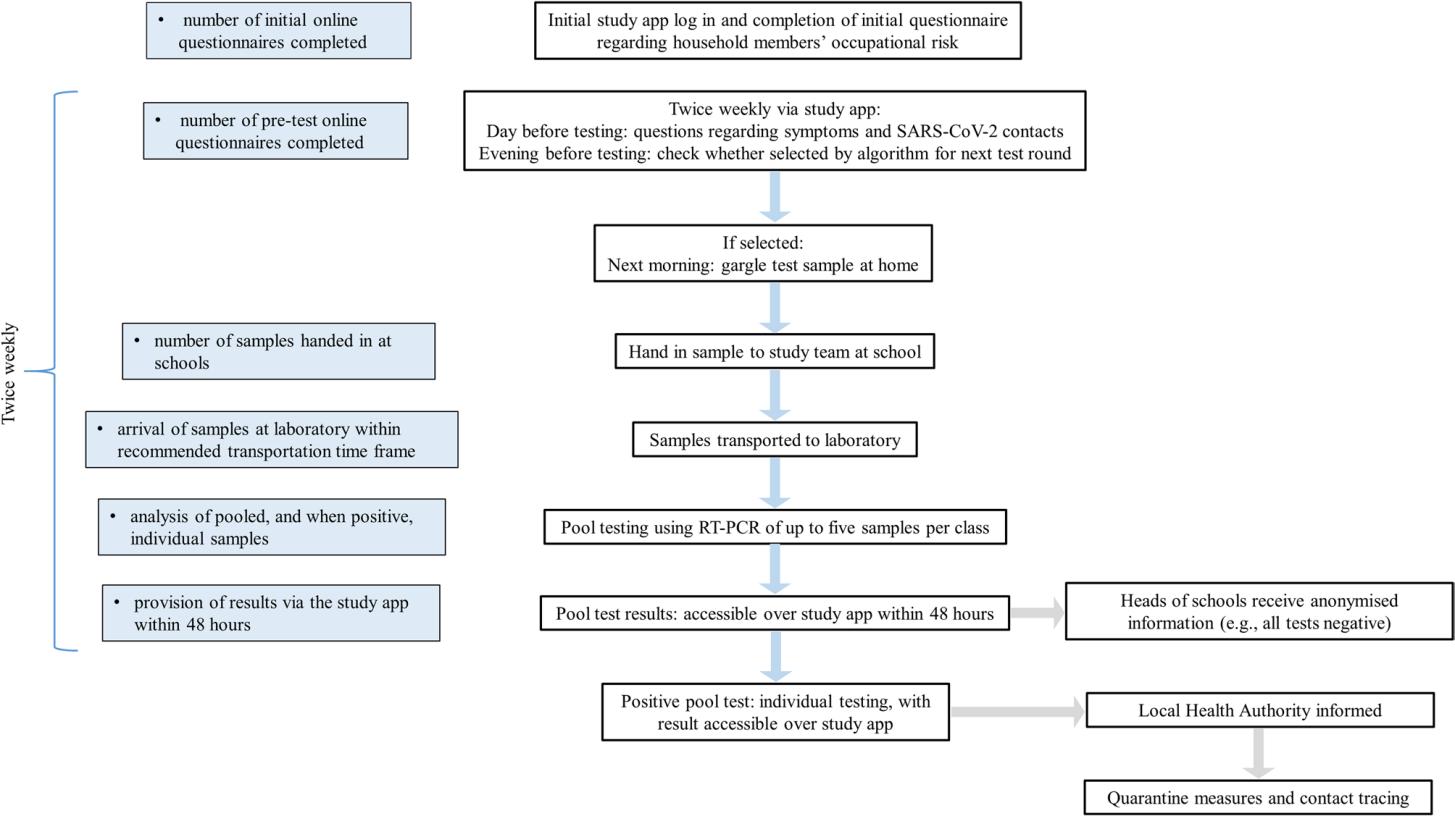
Surveillance programme overview. The light blue boxes indicate the formally evaluated logistical steps for determining programme feasibility.

A detailed study protocol describing the surveillance programme and a brief local summary report in German are available^18,32^. An overview is provided here (Fig. 2). Briefly, on inclusion, pupils received a 16-digit study participation code, a sample collection kit, and written instructions for the web-based study app and sample provision. To preserve anonymity, no record was made of individual code assignment. Participants were asked to enter their code into the study app to answer the Initial Questionnaire (Q1) regarding whether household contacts work in a health or social care setting. Twice weekly, participants were asked to re-enter their code and answer a Pre-Test Questionnaire (Q2) regarding recent symptoms and confirmed SARS-CoV-2 contacts. This information was used to weight the pseudorandom selection of participants for the next test round to maximise the probability of detecting SARS-CoV-2 positive cases. That evening, participants could see via the app whether they had been selected to provide a gargle sample the next morning. This participant selection process was carried out twice weekly, so that the pool of five participants from each class differed for each sample collection. The algorithm was weighted to increase the likelihood of detecting positive cases by computing a priority score based on the variables collected in the Initial (Q1) and Pre-test Questionnaires (Q2) (Fig. 1). As no robust empirical data on the conditional probabilities of each symptom, much less their joint probabilities, was available at the time of study design, weights were assigned based on expert opinion, taking into account available data from the literature and local statistics. Testing was prioritised based on the priority score, i.e., individuals with scores above the threshold (T) were always tested, and the remaining testing slots were assigned randomly.

Gargling of 10 ml of 0.9% NaCl-solution for 5–10 s was performed the next morning in the participant’s home before eating or drinking. No risk of injury is associated. To ensure that the gargle sampling was performed correctly, a detailed, illustrated instruction leaflet was provided to all participants. Study team members received the samples directly from the pupils in the schools and were available to answer any questions. The sample was transferred to a vacuum tube (Vacuette) with a bar code. The participants scanned the bar code using a smartphone camera and the study app to link their personal 16-digit code to the sample, so they could later retrieve their test results via the app. The vacuum tube was labelled with the participant’s name and school, to enable laboratory staff to contact the Local Health Authority if the result was positive. The identification was a legal requirement to enable contact-tracing and potential quarantining. The study team retained no record of individual participant assignment to sample tubes, fulfilling stringent data protection standards. Samples were collected in a reserved room in each school by study staff and prepared for transport to the commercial laboratory (Synlab) at room temperature for real-time reverse transcription-quantitative-PCR (rRT-qPCR) testing using the TaqPath COVID-19 CE-IVD RT-PCR kit (Thermo Fisher Scientific). Regions of the SARS-CoV-2 genome that are not present in other coronaviruses are targeted. RNA was extracted from each sample individually, using an automatic extraction system applying magnetic bead technology (Bio-Rad Laboratories, Magmax, Thermo Fisher Scientific), and pooled for analysis according to class. RNA extraction required around 2 h and the pool testing a further 2 h, consistent with other reports^33^. Pools contained between 1 and 5 samples, although the procedure can be applied to up to 7 samples in a pool. Five pupils could only be selected from a class for a pool if at least 5 Pre-test Questionnaires (Q2) were completed for that test round. Also, fewer than 5 samples were included in a pool if not all the selected pupils remembered to hand in a sample. Samples were to be immediately analysed individually in the case of a positive pool test. The individual results were to be made available over the study app via the 16-digit code within 48 h.

### Evaluation criteria

**Figure 3:**
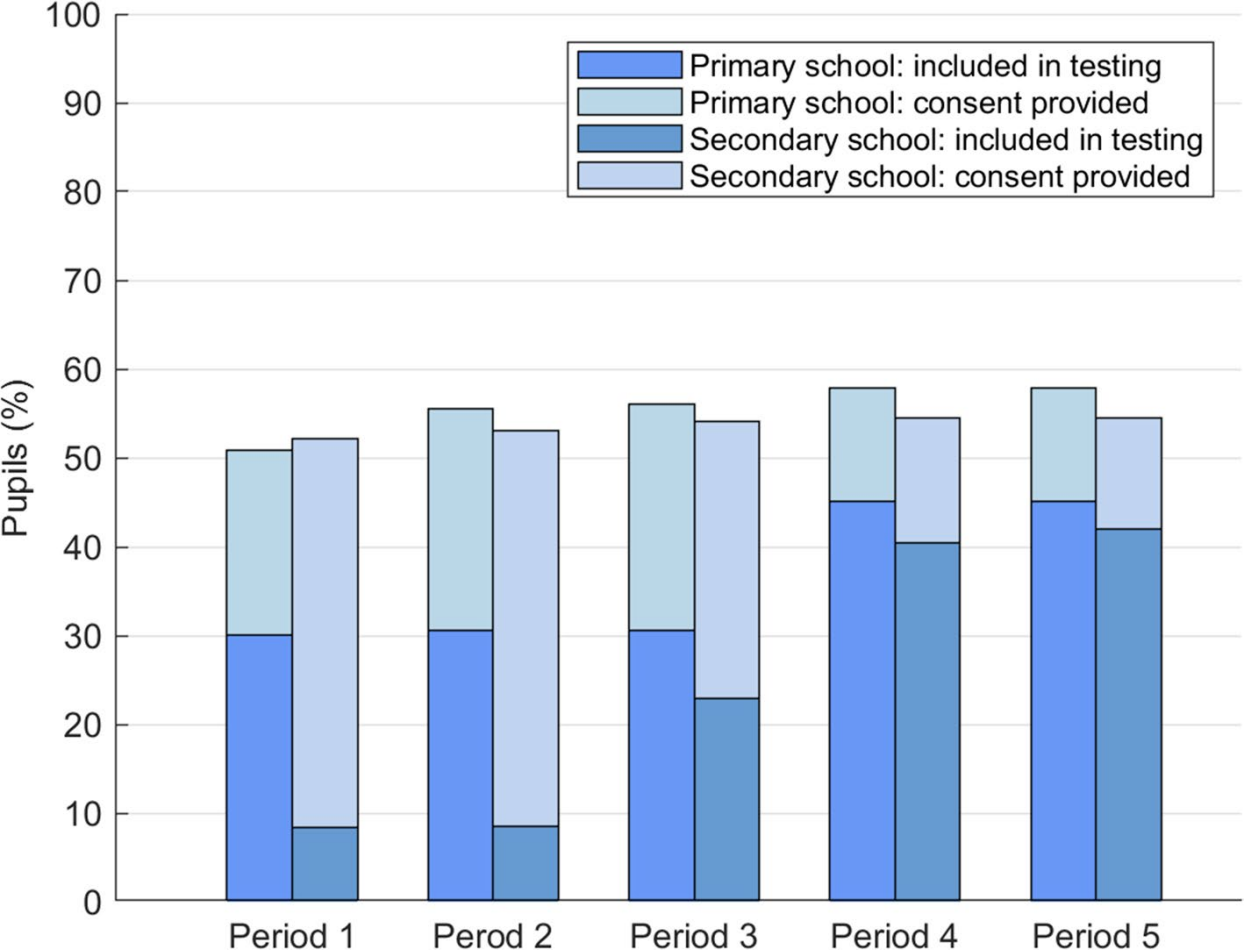
Consent to participation and inclusion in testing. Inclusion initially depended on reaching the 60%, then from Period 4, 50% minimum participation rate in the class. The darker shades of blue indicate inclusion in testing, and the lighter shades of blue indicate provision of informed consent to participate should the minimum participation rate for the class be reached. The bars on the left for each Period represent the primary school and the bars on the right the secondary school.

The programme was evaluated in five periods, defined according to school closure episodes and inclusion of additional classes (Table 1, Fig. 3). The study began in December, 2020, with 8 classes, but was suspended when rising SARS-CoV-2 incidence led to school closures (Period 1: 2–14 December, 2020, 4 test rounds). Testing resumed when schools reopened in March, 2021 (Period 2: primary school, 1–16 March, 5 test rounds; secondary school, 8–16 March, 3 test rounds), 1 week later in the secondary school, due to immediate introduction of rapid antigen testing, temperature measurement, and attendance on alternating days. By mid-March, a further 7 classes (four sixth, one seventh, and two eighth grade classes) reached the 60% threshold for inclusion, taking the number of participating classes from 8 to 15. This test period ended with the start of the Easter holidays (Period 3: 17–26 March, 3 test rounds). When the schools re-opened, the decision was taken to lower the minimum participation threshold from 60 to 50%, resulting in the further inclusion of two first, two seventh, an eighth, a ninth, and two tenth grade classes, as well as the twelfth grade, resulting in 23 participating classes plus the twelfth grade (Period 4: 6 April–5 May, 9 test rounds). The final test period comprised the remaining weeks of the total study period, in which a further tenth grade class could also be included (Period 5: 26 May–16 June, 7 test rounds).

**Table 1:** Summary of participation rates in the testing programme and response rates to the acceptance questionnaires.

Grade	Pupils	Consent provided		Classes included in testing					Questionnaire 3		Questionnaire 4	
		Abs	Rel (%)	Period 1	Period 2	Period 3	Period 4	Period 5	Abs	Rel (%)	Abs	Rel (%)
1	47	25	53.2	0/2	0/2	0/2	2/2	2/2	17/47	36.2	4/47	8.5
2	41	19	46.3	0/2	0/2	0/2	0/2	0/2	14/41	34.1	0/41	0,0
3	47	30	63.8	2/2	2/2	2/2	2/2	2/2	5/47	10.6	6/47	12.8
4	38	23	60.5	2/2	2/2	2/2	2/2	2/2	9/38	23.7	2/38	5.3
5	98	71	72.4	4/4	4/4	4/4	4/4	4/4	47/98	48.0	41/98	41.8
6	108	70	64.8	0/4	0/4	4/4	4/4	4/4	33/108	30.6	49/108	45.4
7	110	60	54.5	0/4	0/4	1/4	3/4	3/4	37/110	33.6	43/110	39.1
8	105	53	50.5	0/4	0/4	2/4	3/4	3/4	43/105	41.0	32/105	30.5
9	109	47	43.1	0/4	0/4	0/4	1/4	1/4	22/109	20.2	12/109	11.0
10	111	58	52.3	0/4	0/4	0/4	2/4	3/4	24/111	21.6	23/111	20.7
11	93	42	45.2	0/1	0/1	0/1	0/1	0/1	10/93	10.8	12/93	12.9
12	96	49	51.0	0/1	0/1	0/1	1/1	1/1	23/96	24.0	10/96	10.4
N/A		3							6		12	
Total	1003	550	54.8	120/521 23.0%	124/536 23.1%	243/547 44.4%	404/552 73.2%	425/550 77.3%	290	28.9	246	24.5

“Consent provided” indicates provision of written, informed consent for inclusion in the testing programme. “Classes included in testing” indicates that the minimum positive response rate threshold was reached, enabling inclusion of the class in the testing programme. Questionnaires 3 and 4 were distributed to all families, irrespective of participation in the testing programme. (Q1 and Q2 were employed as a part of the testing programme to select participants with an increased risk of infection.) Period 1: 2 to 14 December, 2021; Period 2: 1 (primary school) or 8 (secondary school) to 16 March, 2021; Period 3: 17 to 26 March, 2021; Period 4: 6 April to May, 2021; Period 5: 26 May to 16 June, 2021. “abs” = absolute (numbers of pupils). “rel” = relative (to the number of pupils in the class).

The positive response rates for inclusion in the testing programme were examined, and the rates of successful completion of the logistics steps were evaluated individually (Fig. 2). The acceptance of the surveillance programme was assessed according to positive response rates and the answers to the two acceptance questionnaires, which were distributed to all pupils, irrespective of participation in the surveillance programme (see Supplementary information). Q3, addressing reasons for choices over participation, was distributed in paper and electronic form 2 weeks after the study began. Q4, addressing the test method, was distributed in electronic form at the end of Period 3. Questions could be left unanswered. Responses to open questions were openly coded, and a category system was developed, enabling quantification of the expression of individual responses. The resulting codes are presented in the Results section, together with the number of responses in each category.

### Statistical analyses

As the study concept was evaluation of feasibility, exploratory analyses of descriptive statistics were performed. Inferential statistical tests were then applied to assess the significance of observed effects. First, the positive response rates and logistics were assessed (including completion of Q1 and Q2). We then evaluated the responses to the acceptance questionnaires (Q3 and Q4).

Pearson’s chi-square (χ^2^) homogeneity tests were applied to assess whether the positive response rates differed between the schools, and also to investigate specifically whether the pupils’ grade (1–12) had an influence on positive response rates. For each grade, a binomial test was then applied to evaluate whether the positive response rates for each grade were consistent with the positive response rate distribution across all grades.

We applied a logistical regression using IBM SPSS Statistic, Version 26, standard configuration (GENLIN) to evaluate the influence of potential predictors of completion of the participant-dependent logistical steps. The dependent variable was dichotomous and indicated whether the step was completed or not. The potential predictors evaluated were the *Logistical Steps* (completion of Initial Questionnaire, Pre-Test Questionnaire, Sample Handed In) and the *Periods* (5 Periods).

Qualitative analyses and descriptive statistics were applied to evaluate the responses to the acceptance questionnaires (Q3 and Q4), and 95%-Clopper-Pearson confidence intervals were calculated for proportions.

### Ethical statement

The study protocol was approved by the Local Ethics Committee of the University Hospital, Magdeburg. The study was performed in accordance with the ethical standards as laid down in the 1964 Declaration of Helsinki and its later amendments. Participation was voluntary and conditional on provision of informed, written consent from the parent and/or legal guardian of the pupil or the pupil themselves, depending on age. Participants could withdraw from the surveillance programme at any time without providing reasons. The study complied with strict EU and German data protection regulations.

## Results

### Participation rates across schools and grades

Study information was distributed to 1003 pupils. Informed, written consent to participation was initially returned by 520 pupils (51.8% CI_0.95_: [48.7%, 55.0%]) and by the end of the study by 550 pupils (mean across grades: 54.8% [95% CI: 49.2 60.4]). Positive responses rates did not differ between the two schools (χ^2^(1) = 0.20: p = 0.66). The highest participation rate was in the fifth (71 of 98 pupils: 72.4% CI_0.95_: [62.5%, 81.0%]) and the lowest in the second grade (19 of 41 pupils: 46.3% CI_0.95_: [30.1%, 62.6%]), and the positive response rate differed across the grades (χ^2^(11) = 31.0, p = 0.001). The positive response rates in grades 5 (p < 0.001) and 6 (p = 0.02) were higher, while those in grade 9 (p = 0.011) and 11 (p = 0.044) were lower than those expected based on the positive response rates across all grades. However, after Bonferroni correction for multiple comparisons, only the difference in grade 5 remained significant.

Interim evaluation showed that only 26.2% of those providing informed consent to participation could actually be included in the testing programme, based on the 60% threshold having been reached in their class. This consideration, along with numerous enquiries from those families still awaiting inclusion, led to lowering the threshold to 50% for each class. Application of the 50% threshold for starting testing resulted in inclusion of 42.4% of all pupils in testing, thus including 77.5% of pupils for whom informed consent was provided (Fig. 3). The initial threshold was set for STACADO, based on plans for imminent implementation of the programme for infection control purposes. 60% participation was deemed necessary for outbreak prevention. The threshold was adopted by STACAMA to enable direct comparability. STACAMA is a feasibility study to evaluate the acceptability and logistics of the programme in day schools, in contrast with STACADO, in which the model school was a boarding school, however. A 50% threshold enabled preservation of participant anonymity, as it would not be apparent who had received a positive test result, when half or more of the pupils in the class were being tested, while increasing the sample size for robust evaluation of the logistics and acceptance.

### Participation rate across logistical steps and time periods

**Figure 4:**
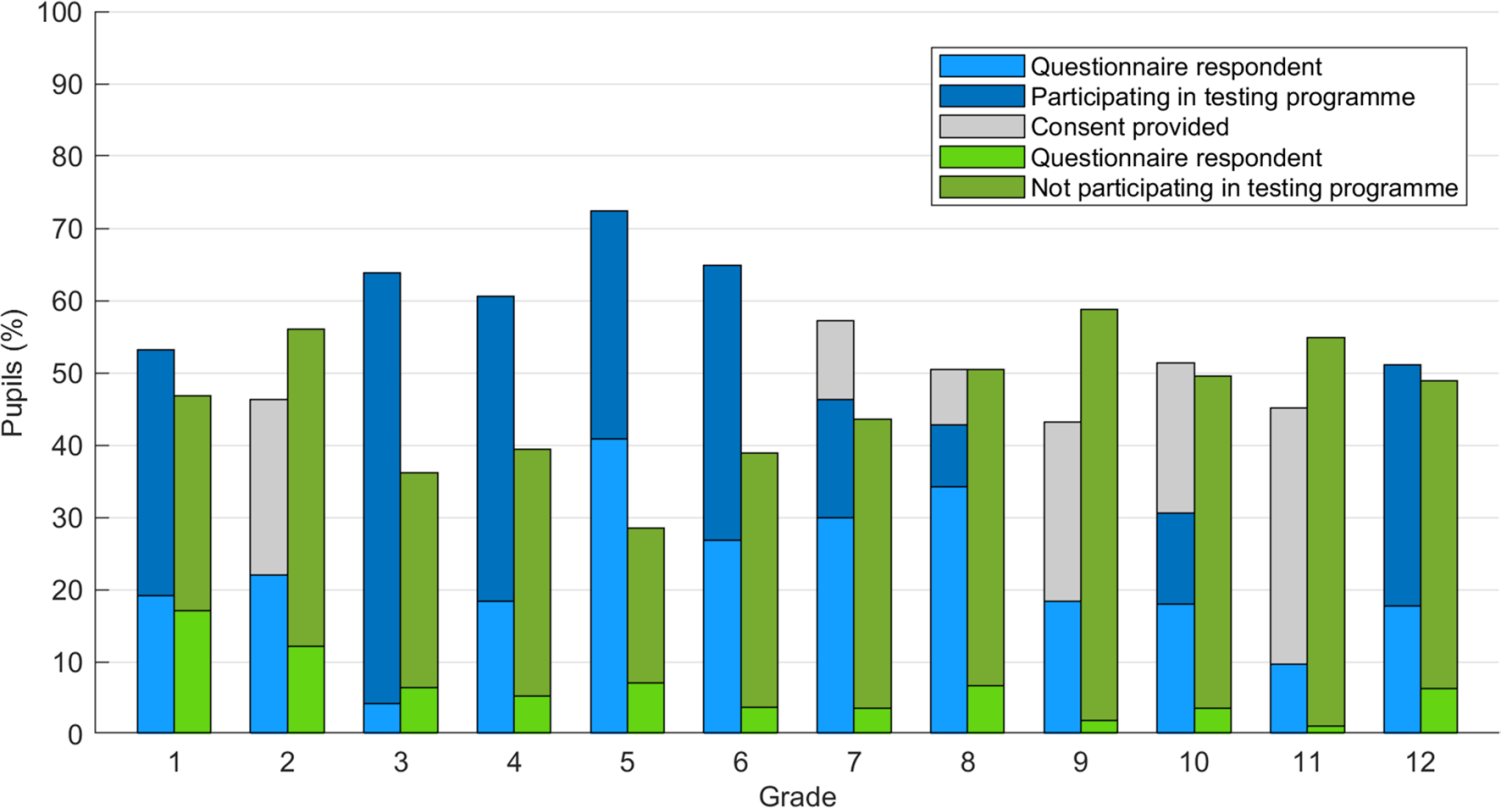
Positive response rates for participation in the testing programme and rates of responding to Questionnaire 3 (Q3) across the school grades, in which reasons for the decision over whether to participate in testing were investigated. The blue bars indicate participation in Q3 by parents and/or legal guardians providing consent to participation in the testing programme, and the green bars indicate participation in Q3 by parents and/or legal guardians not providing consent to participation in the testing programme. Gray indicates the pupils for whom consent was provided, but the minimum participation threshold was not reached for the class.

The Initial Questionnaire (Q1) was completed via the study app at the beginning of the study by 94.2% CI_0.95_: [88.4%, 97.6%] of participants (Fig. 4). The reductions in the percentages given for completing Q1 in Periods 3, 4, and 5 resulted from inclusion of additional classes, in which not all those consenting to participation in the testing programme subsequently filled in Q1 via the study app.

**Figure 5:**
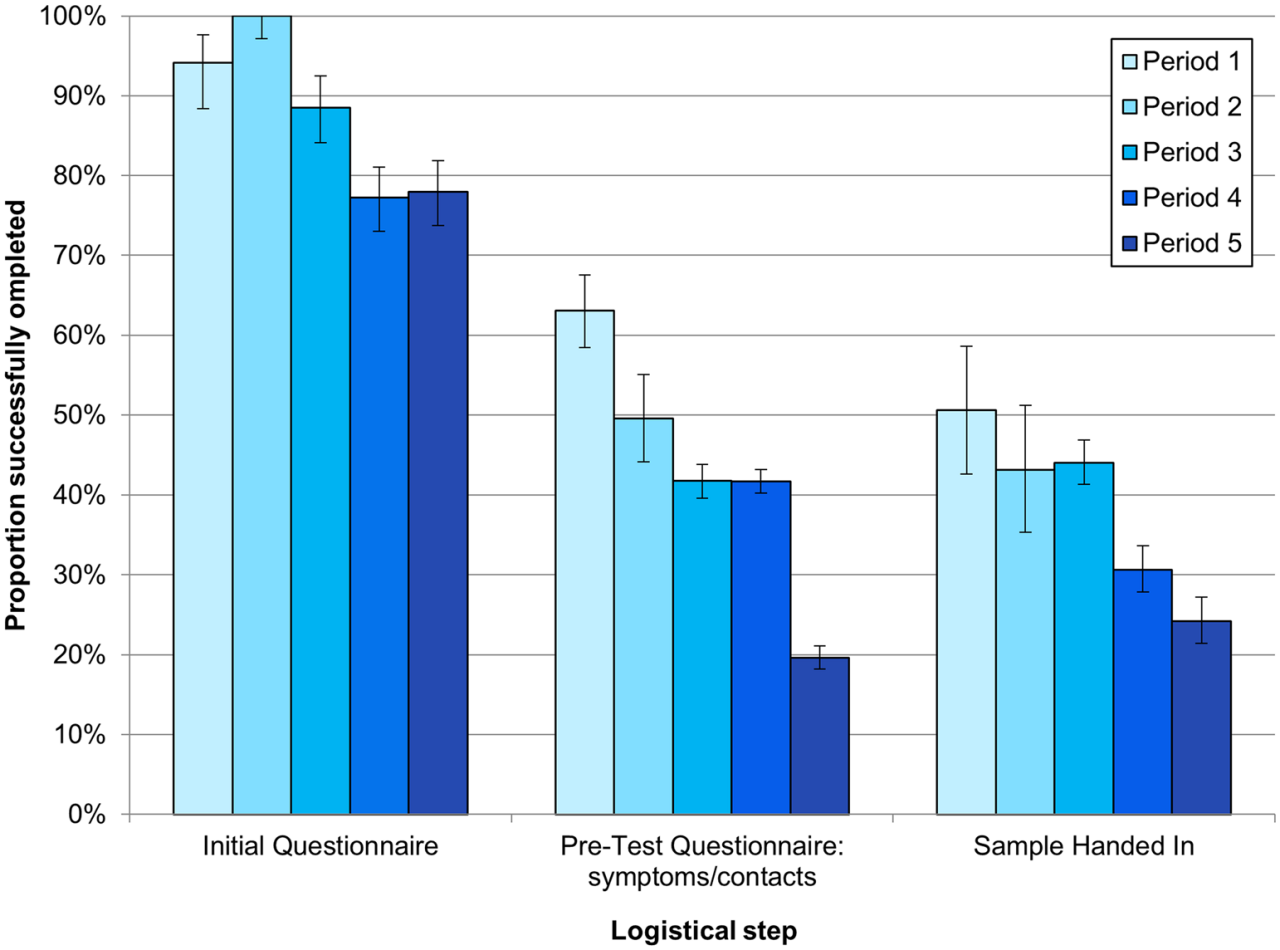
Logistics after inclusion in the study in each data collection period. Period 1: 8 classes in December, 2020, before renewed school closures; Period 2: 8 classes on school re-opening from early March, 2021; Period 3: late March, 2021, after inclusion of 7 additional classes on reaching 60% participation threshold; Period 4: after inclusion of 8 additional classes and the twelfth grade after Easter, 2021, after lowering the inclusion threshold to 50%. Period 5: after additional of a further additional class. The percentages were calculated according to the total expected numbers, based on the number of participating classes and tests in the period, with five samples expected per class.

The omnibus hypothesis that there is no relation between the categorical factors and the successful completion of the participant-dependent logistical steps was rejected (likelihood ratio χ^2^ (6) = 1862.5, p < 0.001). An effect was indicated for both the *Logistical Steps* (Wald χ^2^ (2) = 865.2, p < 0.001) and the *Periods* (Wald χ^2^ (4) = 588.4, p < 0.001). The odds ratios are provided in Table 2. The number of successful completions of the logistical steps decreased both over the *Logistical Steps* and over the *Periods* (Fig. 5).

**Table 2:** Wald ${\chi }^{2}$, p-values, and odds ratios for the factor levels in the logistic regression model for the successful completions of the logistical steps.

Predictors	Wald ${\chi }^{2}$	p-value	Odds ratio and 95% Wald CI		
			Point estimates	Lower bound	Upper bound
Constant	668.9	< 0.001	0.236	0.211	0.263
Initial questionnaire (Q1)	767.0	< 0.001	11.449	9.635	13.605
Pre-test questionnaire (Q2)	10.8	0.001	1.186	1.071	1.314
Sample handed in	–	–	1	–	–
Period 1	374.5	< 0.001	5.587	4.694	6.650
Period 2	197.1	< 0.001	3.745	3.115	4.503
Period 3	183.6	< 0.001	2.751	2.376	3.185
Period 4	240.0	< 0.001	2.219	2.006	2.455
Period 5	–	–	1	–	–

### Further logistics

All test samples that were handed in arrived at the laboratory within the required time for processing. The test results were available over the study app within 48 h of sample collection for transport. In total, 792 tests were carried out for the two schools, of which one was positive.

### Gargle test results

The weekly incidence in the study region ranged between 5.2 and 182.7 per 100,000 population during the study period^34^. Therefore, in a class of 25 pupils, between 0.0013 and 0.046 cases would be expected in a class in a given week. Over the 16 weeks of testing, the number of expected positive cases in a class was therefore between 0.021 and 0.73. The detection of a single positive case in the current study is thus in keeping with the expected detection rate.

### Acceptance questionnaires

**Figure 6:**
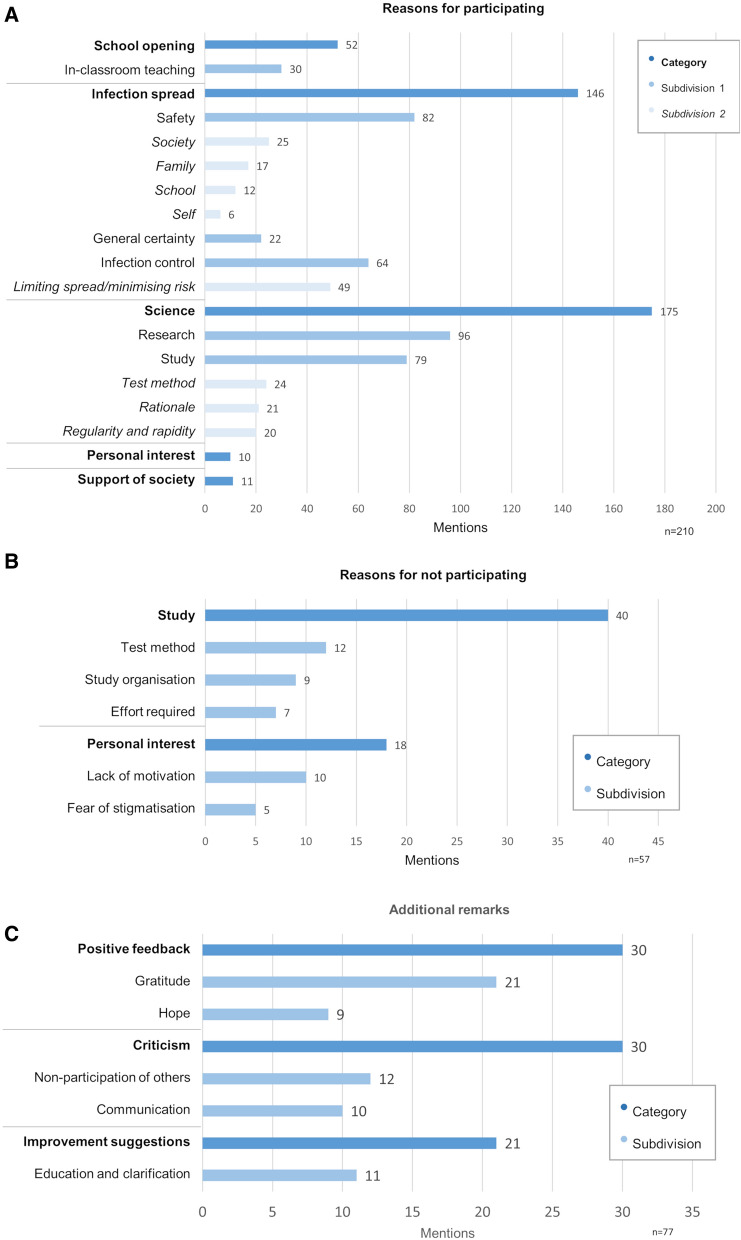
Reasons given in Questionnaire 3 for participants' decision over participation. (**A**) Reason given for participating in the testing programme. (N = 210 respondents). (**B**) Reasons given for choosing not to participate in the testing programme. (N = 57 respondents). (**C**) Additional remarks provided by respondents. (N = 77 respondents).

Q3 was completed by 290 pupils with their families (28.9% response rate), of whom 232 pupils (80.0%) were participating in the testing programme. 85.9%´of participation decisions were made by the pupil and family together, and 96.4% were in agreement. Reasons for participation were provided by 210 participants (79.2% of Q3 respondents) (Fig. 6A). We focussed on responses provided by at least five respondents. The reasons could be categorised as follows: Science, Infection Spread, School Opening, Society, and Personal Interest. Science was named most frequently, by 83.3% of respondents. Infection Spread was named by 69.5% of respondents. A response in the School Opening category was given by 24.8% of respondents. The remaining categories included more individual responses, including supporting society in general (5.2%) and personal interest, including in own results (4.8%). Reasons for not participating were provided by 57 (19.6%) respondents, including one respondent participating in the testing programme (Fig. 6B). Those provided by at least five respondents could be categorised as Study (referring to support for the study as a concept, as well as to practical aspects of the study design) and Personal Interest. Additional remarks were provided by 77 (26.6%) respondents (Fig. 6C). Positive and critical remarks were equal in number (N = 30). The hope expressed was that the study would continue, or that new testing programme participants could start after the lockdown. Criticism included an expression of lack of understanding of those unwilling to participate, in association with a perceived minimal effort required to participate or an absence of disadvantages from taking part.

**Figure 7:**
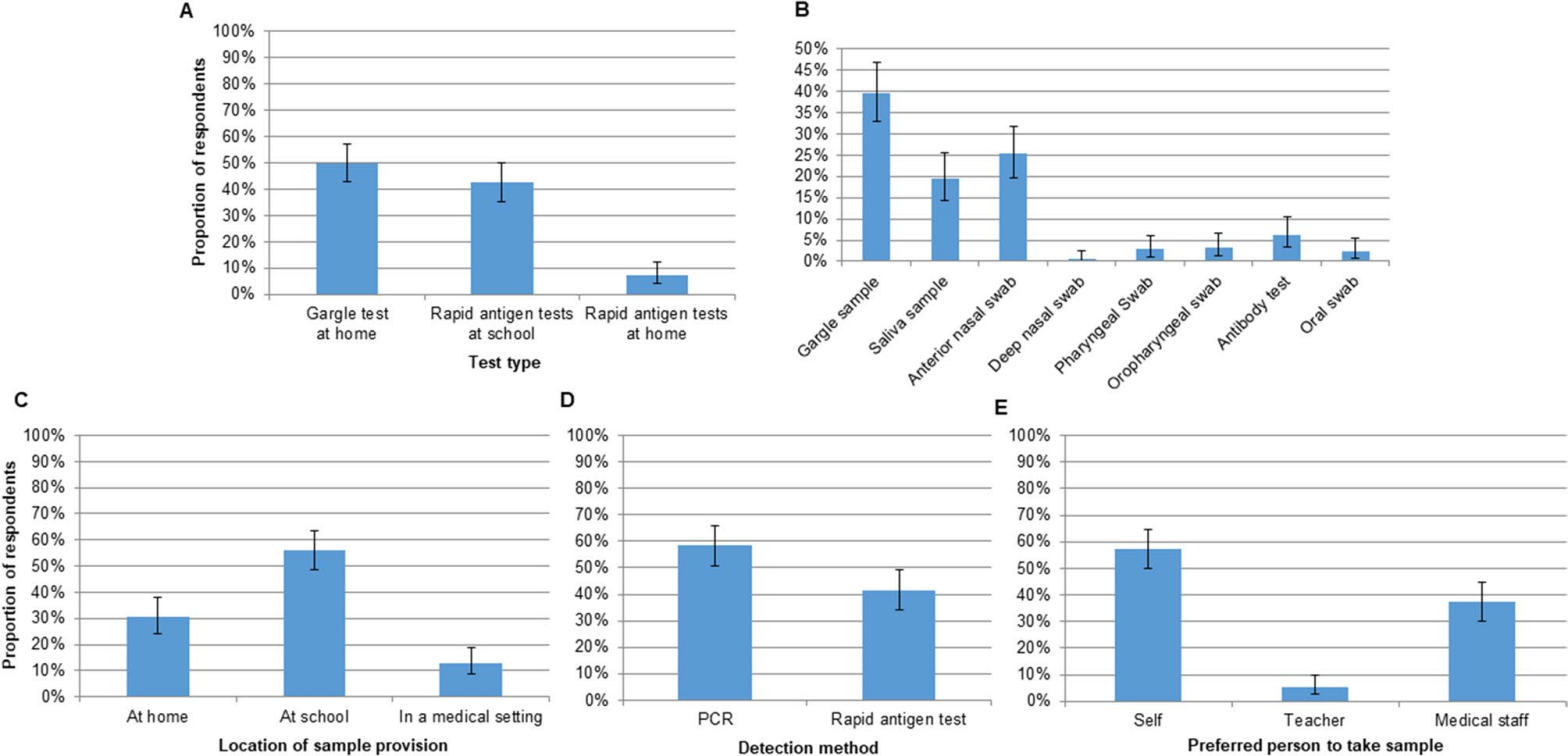
Responses to Questionnaire 4, in which participants were provided with multiple choice options regarding SARS-CoV-2 testing. (**A**) Reasons for favouring a particular SARS-CoV-2 test type (N = 188). (**B**) Choice of sampling method for SARS-CoV-2 testing (N = 209). (**C**) Choice of location for provision of test sample (N = 185). (**D**) Choice of SARS-CoV-2 detection method (N = 169). (**E**) Preference of a particular person to take test samples (N = 185). Error bars: 95% CI.

Q4, focussing on the test method, was completed by 235 respondents (23.4%), of whom 217 (92.3%) had provided informed consent to testing programme participation and 145 pupils (61.7%) were involved in testing. Examining *test type*, slightly more respondents preferred the gargle test at school than the rapid antigen test at home (Fig. 7A). Reasons given for a preference for the former included ease of sample collection (N = 33) and result validity (N = 19). Ease of use (N = 15), test location (N = 10), and short time for results (N = 9) were named in favour of the latter. Choosing between *types of sampling* (Fig. 7B), the most frequently chosen were the gargle sample, the saliva sample, and the anterior nasal swab. Examining preference for a *particular location for sample provision* showed the highest number of respondents preferring sample provision at school (Fig. 7C). Choosing between *types of detection method*, more respondents expressed a preference for laboratory-based PCR test than for the rapid antigen test (Fig. 7D). Finally, examining preferences for a *particular person to take the sample*, the highest number of respondents would prefer for the child to take the sample themselves (Fig. 7E). Details of the reasons behind these choices are provided in Supplementary information (Figs. S1–S5).

## Discussion

We evaluated the acceptability and the logistics of a SARS-CoV-2 surveillance programme in two schools in Magdeburg, Germany based on gargle samples and PCR-based pool testing. The aim was to establish whether the programme could provide a feasible means of preventing outbreaks and potentially ensuing school closures. We chose gargle sampling as it is non-invasive, and PCR-testing provides high sensitivity and specificity^20^. Sample collection at home avoids infection spread through testing in schools. A high acceptance of the approach was indicated by positive feedback through the questionnaires. In the context of the feasibility study, schools remaining open was not dependent on the surveillance programme, and participation involved the risk of receiving a quarantine order despite a potentially symptom-free infection. Given these factors, the participation of over half of the pupils was deemed high.

Evaluation of the logistics of the programme provides support for its feasibility if implemented school-wide, as the limiting factors in the logistics could be addressed by reminders, if all pupils were participating in the programme. Twice-weekly compulsory testing, as a prerequisite for attendance, has indeed already been introduced into schools in Germany using rapid antigen tests, with provision of homeschooling for those not willing to be tested. Over three-quarters of families providing informed consent to testing programme participation successfully completed the Initial Questionnaire in the study app, suggesting the approach is user-friendly. The critical steps in the logistics were identified as entering the participant code into the study app for selection for the next test round and handing in of the sample at school, as significantly fewer participants completed these steps than completed the Initial Questionnaire. The voluntary participation, together with stringent data protection measures, limited the potential for sending reminders. Introduction of the surveillance programme school-wide would enable implementation of reminder systems for filling in the twice-weekly Pre-Test Questionnaire and handing in samples. Samples were not collected in the classrooms in order to preserve anonymity, requiring pupils to remember independently. Secondary school pupils could, moreover, potentially complete the Pre-Test Questionnaire at school; if the surveillance programme included all pupils, anonymity would be preserved through the high numbers of samples collected. A further consideration is the reduced school attendance to alternating days following the lockdown due to high infection incidence, which is likely to have prevented programme participation from becoming routine for those involved, because pupils alternated the weeks in which they were present in school on the testing days.

The completion rate for the participant-dependent logistics steps reduced over the course of the study. While the reasons for not handing in a sample are likely to be many and varied, we postulate that the reduction in the proportion of expected samples actually received could reflect a decline in the motivation levels of pupils and their families to remember to perform each step. It is also plausible that the independent introduction of compulsory rapid antigen testing in the schools during the course of the study period meant that families no longer considered active participation to offer any benefit.

The ensuing logistical steps (transport, analysis, communication of results: see Fig. 2) were successfully implemented, and the individual test results were available over the study app within 48 h, supporting the feasibility of a programme involving sample transport and laboratory testing. The time taken for provision of test results requires consideration, however. The programme was designed for the surveillance of asymptomatic children based on sampling a selected group each time rather than all the pupils in a class. Receiving the results 48 h later for the particular children selected is sooner than obtaining results for children not selected for the particular test round. Although rapid antigen testing leads to results within 15 min, a subsequent PCR test is still required in Germany to confirm the results, due to the higher false positive of rapid antigen tests^31,35^. Moreover, any test is only valid at the time of its performance. Indeed, a negative rapid antigen test is currently only deemed valid in Germany for 24 h^35^, in contrast to 48 h for a PCR test^36^. The costs, both financial and in time, of daily rapid antigen testing of all pupils have thus far precluded such an approach. This evaluation is based on low infection incidence levels. It is arguable that more rapid provision of results would be required at higher infection incidence levels. The laboratory analysing the samples for the current study is not local. If pool-testing could be established in a local laboratory, results could be provided, according to the laboratory, within 24 h. The provision of regular population testing presents a substantial challenge to laboratory capacities. Methods are being developed through which increasing numbers of samples can be included in a single pool, while reducing processing times. One such approach is the Cap-iLAMP (capture and improved loop-mediated isothermal amplification) method^33^. It can be applied to pools of up to 25 gargle samples at a reagent cost of around 1 Euro per individual. Detection of a single SARS-CoV-2-positive individual in a pool of 26 samples has been possible, although some loss of sensitivity is recognised. On direct comparison, the sensitivity exceeded that of rapid antigen tests, and the processing time was 55 min in contrast with 4 h for RT-qPCR^33^. Indeed, these developments are also relevant to the cost implications of including all pupils in a testing programme. While pooling increased laboratory efficiency in the context of the current testing programme, the costs per pupil in the pool were nonetheless based on those for individual testing and were around 30 Euros per individual.

Approximately half of those providing informed, written consent to participate in the surveillance programme completed Q3, which addressed reasons behind the decision over participation, of whom 80% were testing programme participants. The most frequently given reason for participation was an interest in supporting science, including research in general and the study in particular. Limiting infection spread and keeping the schools open were also frequently named. These reasons suggest a willingness to engage actively in finding ways to keep schools open, while protecting the vulnerable. The most frequently reported reason for not participating was the test method used, and we examined this aspect further in the subsequent Q4. We interpreted the relatively low participation rate in Q3 among those not enrolled in the programme as suggesting a general rejection of participation in any aspect of the study rather than specific objections to the testing programme, because specific objections could have been communicated through the anonymous acceptance questionnaire. In the majority of cases, the children/young people and their parents and/or legal guardians agreed over participation.

Diverse test strategies have been implemented in schools to detect SARS-CoV-2 since the first school closures in Spring, 2020. Participation rates were notably higher when gargle^37^ or saliva^38^ samples were employed than when oral^30^ or nasopharyngeal^39^ swabs were taken. Gargle samples were provided for pool testing by 63% of pupils in a study in Austria, before renewed school closures^37^ and by 83% of pupils in STACADO. Self-collected saliva samples were PCR-tested for a primary school in Norway to investigate viral transmission from confirmed cases^38^. The high participation rate, at 73%, may be explained by the non-invasiveness of the sample collection. By contrast, mouth/throat swabs, taken by study personnel for PCR-analysis, were offered in Zurich, with an uptake rate of 49%^30^. The high participation rate in providing saliva samples could also be because the target population had had contact with a positive case, however^38^. On the other hand, testing of confirmed contacts over an 11-week period in schools and kindergartens in Australia using nasopharyngeal swabs had a participation rate of 44%^39^. Drop-outs were reported for two studies in which nasopharyngeal swabs were taken (0.24%; nearly 5%)^40,41^. We postulate that willingness to participate in surveillance programmes in schools could also depend on the wider consequences of participation. Introduction of a testing programme that enables a school to reopen, after having been closed to reduce infection spread, may be viewed by pupils and their families more positively than introduction of a testing programme in a school that is open and has the potential to be closed should an outbreak, which may have only involved asymptomatic individuals, be identified. It is indeed plausible that this aspect has a greater impact on the acceptance of a surveillance programme than the choice of sample method used for testing. The risk of a personal quarantine order for an asymptomatic child may be regarded as a disadvantage among families without at-risk members or contact with individuals at risk. A further consideration in the choice of test is the sensitivity and specificity. A comparison was performed between the number of positive rapid antigen tests and the number of positive tests through oral swab in the school surveillance study in Zurich^30^. False positive tests were reported for 0.6% of the children and 1.7% of the teachers tested. Acceptance of the procedures was not compared. The authors concluded that individual testing, with low infection prevalence in schools and also considering the false positive tests with the procedures used, was inefficient.

At the beginning of Period 2, subsequent to completion of the first acceptance questionnaire (Q3), twice-weekly rapid antigen testing through self-administered anterior nasal swabs was introduced on a compulsory basis at the secondary school by the school administration and subsequently in all schools by the state. Mandatory participation in the rapid antigen testing meant that all attending pupils were regularly tested, in contrast with STACAMA, in which participation was on a voluntary basis, in accordance with the guidelines in place for study contexts. Participants therefore gained direct experience of two test methods, enabling a direct comparison of acceptability. In Q4, although more respondents expressed a preference for the gargle tests at school than antigen tests at home, the difference between numbers of respondents was small. The test location was among the reasons given in favour of the antigen test at school, and this factor could have led to an inflation of the numbers of respondents choosing this option. Indeed, on specific questioning over preferred location, the highest number of participants preferred samples to be provided at school. Moreover, when specifically questioned over a preference for a particular detection method, more respondents would prefer PCR to rapid antigen testing. Most respondents preferred that the pupil be able to perform sample collection themselves. Responses to the questionnaire addressing reasons over the choice over whether to participate showed a strong interest in supporting science. While a related project week was provided in the primary school, as well as age-appropriate presentations from a member of the Hospital Department of Hygiene at the secondary school, alongside information evenings at each for parent representatives from each class, communication with pupils and their families was limited, due to contact restrictions in place to limit infection spread. Future studies could build on this interest, and potentially further increase participation, by arranging outreach sessions to discuss the programme during its implementation with pupils and their families.

Our findings should be interpreted in the context of the model schools. Both are located in Magdeburg, the capital of the state of Saxony-Anhalt in Germany. The primary school was a state-funded school attended by children within the local catchment area, and the secondary school was partially government-subsidised, with an additional monthly attendance fee. This fee could be covered by the school parents’ society, so that attendance was not precluded on financial grounds. We did not collect individual parameters pertaining to the profile of participants, such as socio-economic or educational status. The study design was finalised in discussion with the Coordination Centre for Clinical Studies and Department for Data Protection, with an emphasis on participant anonymity. The collection of personal socio-economic data was deemed likely to negatively impact on positive response rates. The positive response rates did not differ between the schools in any case, although we note that positive response rates were highest in the fifth grade. The fifth grade is the youngest year group in the secondary school. We speculate that motivation levels were high to support the school remaining open given the pupils’ recent start in a new school, and starting in a new school entails adaption to a multitude of systems new to the pupils and families, potentially increasing openness to the programme.

We note that the aim of the current study was to evaluate the feasibility of the surveillance programme, based on gargle samples and pool testing, according to acceptability and logistics. The relatively low infection incidence in Magdeburg at the time of the study and the number of pupils involved preclude a comparison of sensitivity and specificity between the test in our surveillance programme and the independently introduced programme based on rapid antigen testing. Moreover, while rapid antigen testing was introduced on a compulsory basis and included all pupils, participation in our programme was voluntary and anonymous. As a result, it was not possible to link findings between the two test methods. We note, however, that the detection of a single positive case was in keeping with the expected detection rate based on local infection incidence rates during the study period.

Given the well-known and considerable disadvantages of school closures for children and young people, together with the risk of spread of mutated virus strains with higher infectivity rates, a regular testing strategy in schools is deemed advisable^14,15,31,42^. The high sensitivity and specificity of PCR-based testing using gargle samples^20,25^, the non-invasiveness of sample collection^20^, and the indication from modelling that pool testing provides more efficient case detection than individual testing when infection prevalence is low^29^ suggest that the approach applied here is promising. Other advantages of a laboratory-based test method include immediate individual sample testing if a pool test is positive, and the infrastructure to communicate with Local Health Authorities should quarantining and contact tracing be required. The current study suggests that such a surveillance programme is, moreover, feasible. We observed a high willingness to participate, despite the fact that participating in the current study did not offer direct prevention of school closure, because not all classes could be included. Not testing all classes meant that the risk remained that an outbreak could already involve many asymptomatic pupils before a positive case was detected following development of symptoms. Moreover, evaluation of the logistics enabled identification of challenging steps, namely filling in the Pre-Test Questionnaire and handing in a sample, for which solutions are practicable. Such a programme provides a potential means of preventing infection outbreaks and ensuing school closures both during the current pandemic and in potential future scenarios, in which a highly transmissible respiratory virus results in high morbidity and mortality, and containment of viral transmission is required.

## Supplementary Information

Supplementary Information 1.

Supplementary Information 2.

## Data Availability

The data generated and analysed during the current study are made available within the manuscript itself.
