# Supplementary material for: Feasibility of a surveillance programme based on gargle samples and pool testing to prevent SARS-CoV-2 outbreaks in schools
Source: Sci Rep. 2021 Sep 30;11:19521. doi: 10.1038/s41598-021-98849-1 (PMC8484445; doi:10.1038/s41598-021-98849-1)
Supplement: Supplementary file 1 — Supplementary Information 1. [file 41598_2021_98849_MOESM1_ESM.docx]

**Supplementary figures**


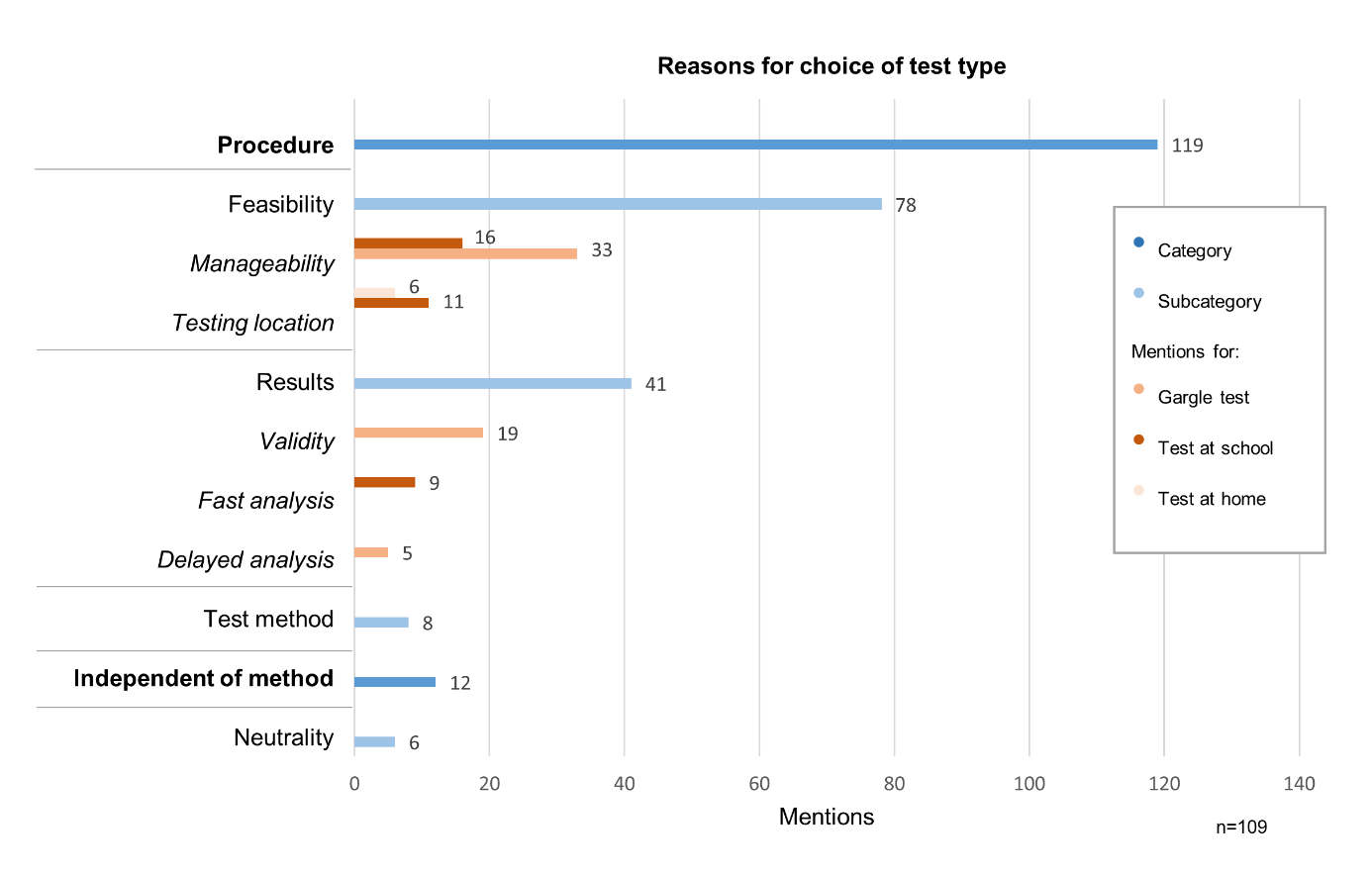


**Fig. S1**. Reasons for favouring a particular test for SARS-CoV-testing provided by respondents to Questionnaire 4.


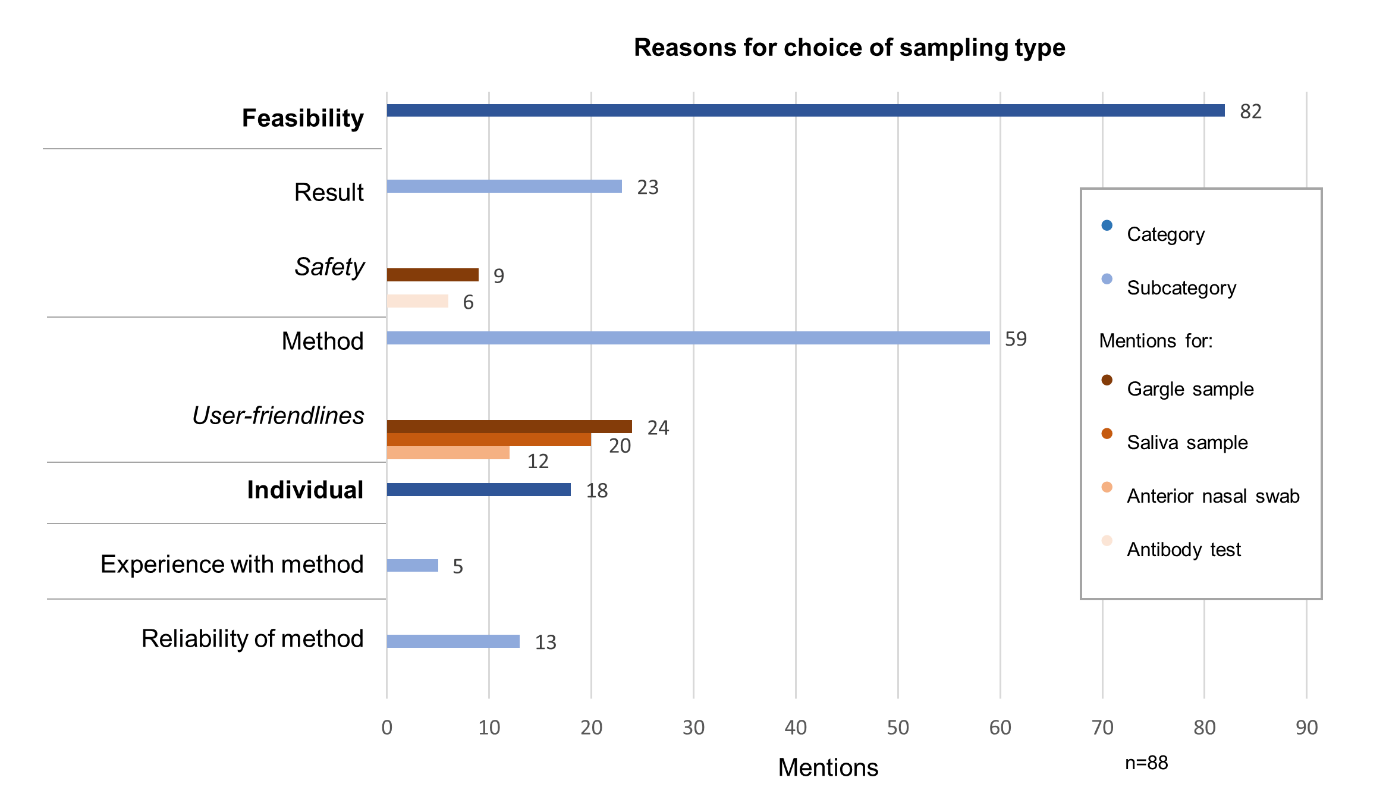


**Fig. S2**. Reasons for favouring a particular type of sample for SARS-CoV-testing provided by respondents to Questionnaire 4.


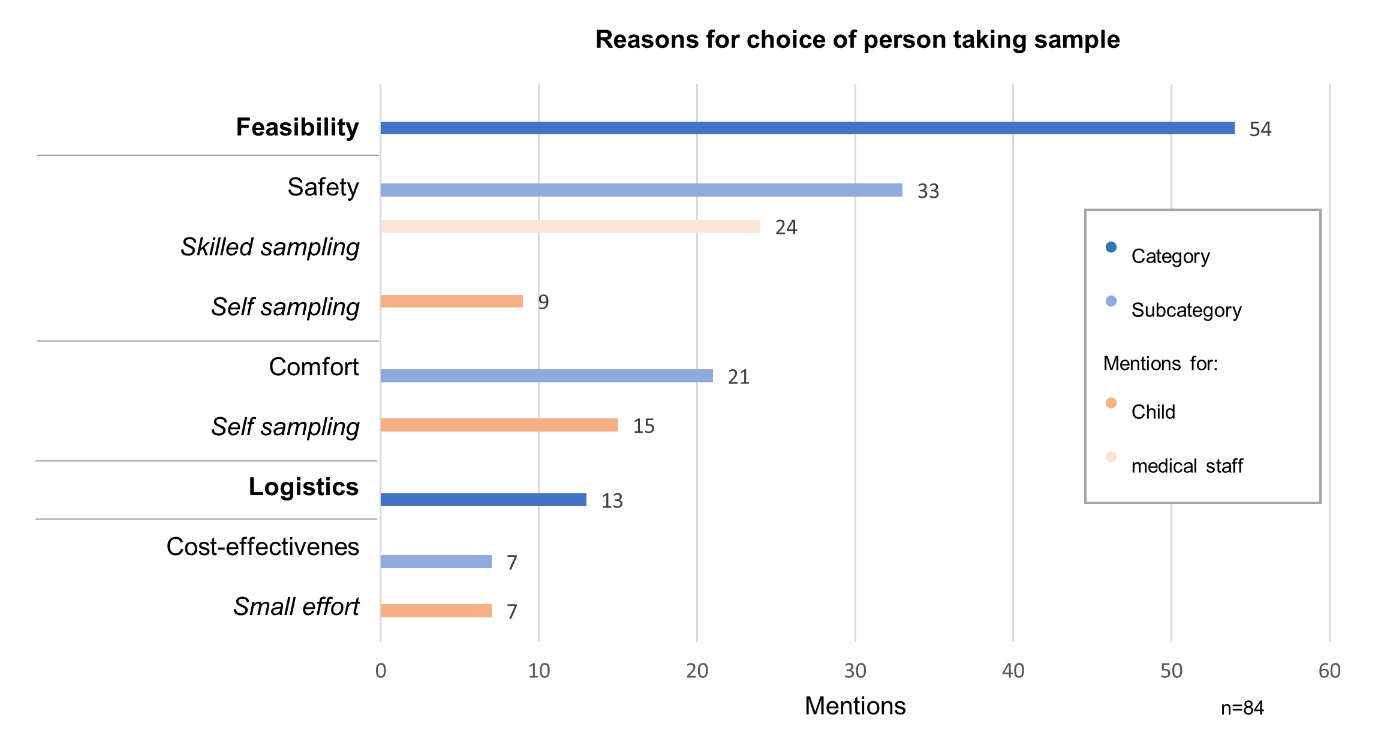


**Fig. S3**. Reasons for favouring a particular person to take a sample for SARS-CoV-testing provided by respondents to Questionnaire 4.


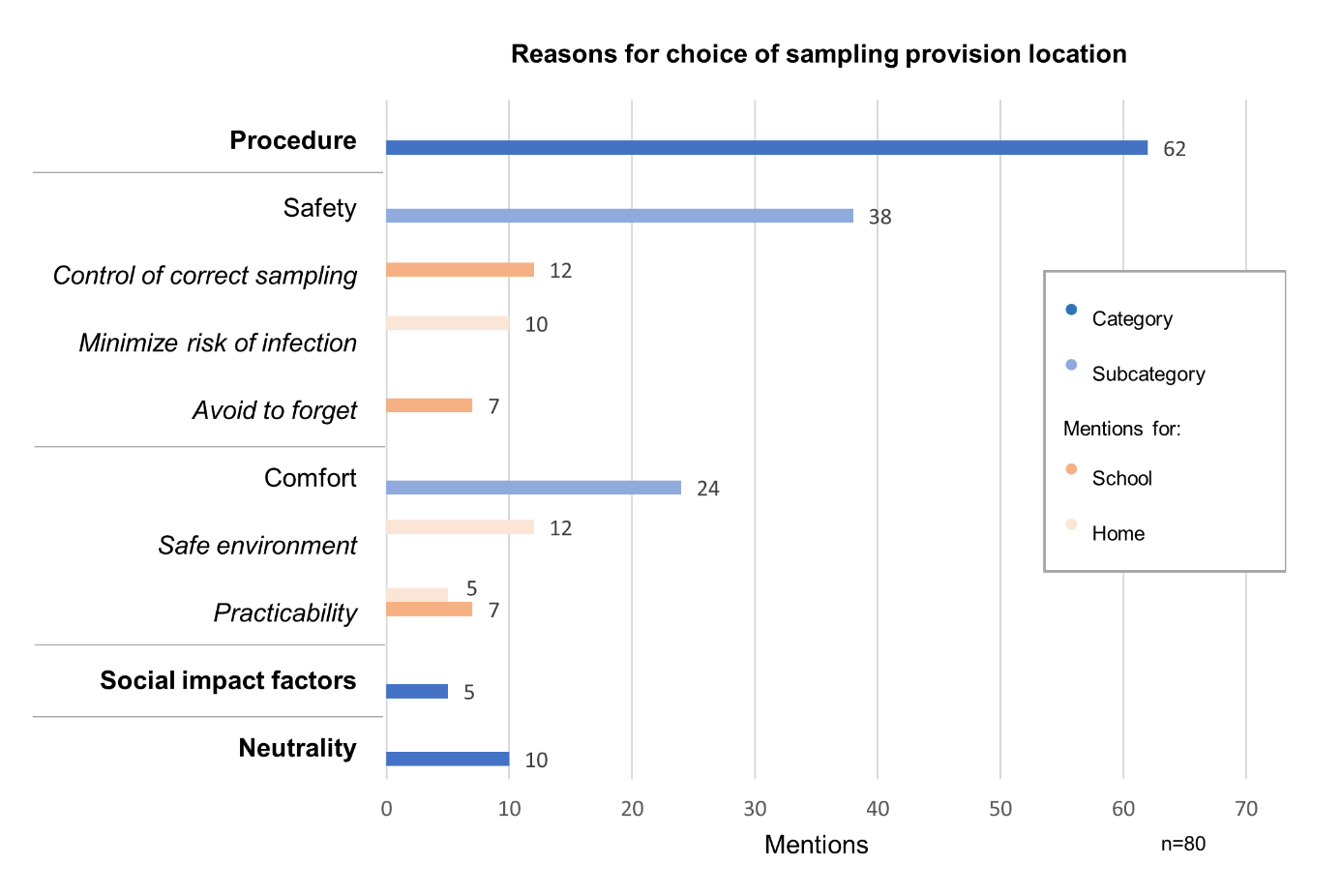


**Fig. S4**. Reasons for favouring a particular type of sample for SARS-CoV-testing provided by respondents to Questionnaire 4.


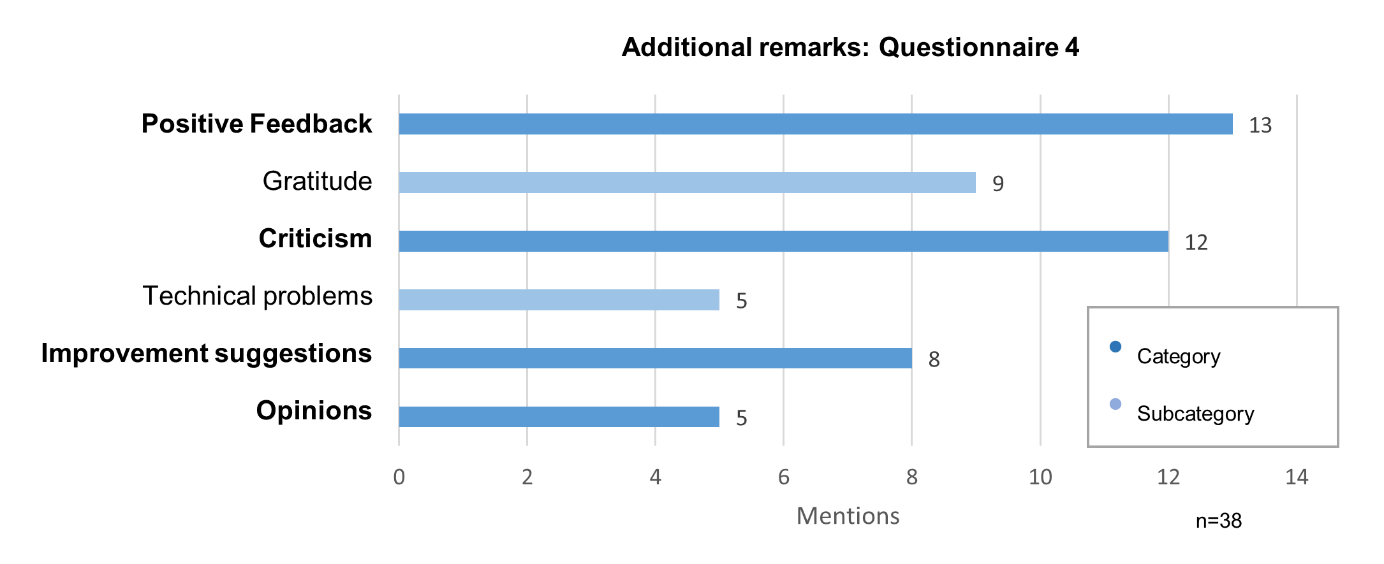


**Fig. S5**. Additional remarks provided by respondents at the end of Questionnaire 4.
