# Supplementary material for: Feasibility of a surveillance programme based on gargle samples and pool testing to prevent SARS-CoV-2 outbreaks in schools
Source: Sci Rep. 2021 Sep 30;11:19521. doi: 10.1038/s41598-021-98849-1 (PMC8484445; doi:10.1038/s41598-021-98849-1)
Supplement: Supplementary file 2 — Supplementary Information 2. [file 41598_2021_98849_MOESM2_ESM.docx]

**Supplementary information**

**Questionnaire 1**. Initial Questionnaire.

| 1. | Wer beantwortet den Fragebogen? | Who is answering the questionnaire? |
| --- | --- | --- |
| 2. | Mir ist bekannt, dass bei dieser Studie Daten, insbesondere Gesundheitsdaten (medizinische Laborbefunde) von den Teilnehmern erhoben, gespeichert und ausgewertet werden. Es werden keine personenbezogenen Daten gespeichert. Eine Verknüpfung zur Person ist an keiner Stelle möglich. Der anonyme Datensatz wird bis zum Ende der Studien-bezogenen Auswertungen aufbewahrt und spätestens am 31.12.2021 gelöscht. Eine Weitergabe von Daten an Dritte zu wissenschaftlichen Auswertungen ist möglich. | I am aware that during the study, data of the participants, in particular health-related data (laboratory findings), will be collected, stored, and analysed. No personal data will be stored. The data are anonymised; It is not possible to link any data to a particular participant. The anonymous data set will be retained until the end of the study-related evaluations and will be deleted on 31.12.2021 at the latest. A transfer of data to third parties for scientific evaluations is possible. |
| 3. | Ich willige ein, dass nach dem Zufallsprinzip bis zu 2x pro Woche für eine Dauer von 16- Schulwochen Rachenspülungen zur SARS-CoV-2 Diagnostik durchgeführt werden. | I consent to participate in the randomly assigned gargling test up to twice per week for 16 school weeks. |
| 4. | Ich bin mit der Beantwortung von Fragen (z. B. zum Alter und zur evtl. CoViD-Symptomatik) einverstanden. | I agree to answer the questions in the study; e.g. about my age and eventually SARS-CoV-2 symptoms. |
| 5. | Ich wurde schriftlich über das Wesen, die Bedeutung, die Vorteile und die Risiken der wissenschaftlichen Untersuchungen im Rahmen der o.g. Studie informiert und hatte ausreichend Gelegenheit, telefonisch und/oder persönlich meine Fragen mit dem Studienteam zu klären. | I have been informed in writing about the nature, significance, benefits and risks of the scientific investigations in this study and had the opportunities to clarify all the question(s) raised by phone and/ or in person with the study team. |
| 6. | Ich habe eine Kopie der schriftlichen Studieninformation und der Einwilligungserklärung erhalten. | I received a copy of the study information and the informed consent form. |
| 7. | Mir ist bekannt, dass die Teilnahme freiwillig ist und das Recht besteht, die Einwilligung jederzeit ohne Angabe von Gründen und ohne nachteilige Folgen zurückzuziehen und einer Weiterverarbeitung der Daten und Proben zu widersprechen und ihre Vernichtung zu verlangen. | I am aware that participation is voluntary and that I have the right to withdraw consent at any time, without giving reasons and without having adverse consequences, and to object the further processing of the collected data and samples and to request to have my data and/or samples destroyed. |
| 8. | Mir ist bekannt, dass Studienteilnehmer das Recht haben, sich bei der unten genannten zuständigen Datenschutzaufsichtsbehörde zu beschweren. | I am aware that participants have the right to complain to the National Data Protection Authority. |
| 9. | Zu welcher Altersgruppe gehört der Teilnehmer? | In which age group is the participant? |
| 10. | Welche Klasse besucht der Teilnehmer? | Which class does the participant attend? |
| 11. | Ist ein Mitglied aus dem gemeinsamem Haushalt beruflich im Bereich der [m]Pflege oder Krankenversorgung[/m] tätig? | Is any household member of the participant employed in the health or social care sectors? |

**Questionnaire 2**. Pre-Test Questionnaire

| 1. | Gab es in der Umgebung z. B. im Freundeskreis, bei Verwandten oder Bekannten (nicht im eigenen Haushalt) eine SARS-CoV-2 PCR positive Person? | Has there been any contact with a SARS-CoV-2 PCR positive person, e.g. among friends, relatives, or acquaintances (outside your own household)? |
| --- | --- | --- |
| 2. | Trat/Traten seit der letzten Befragung Symptome einer Corona-Infektion auf? | Has the participant developed any SARS-CoV-2 symptoms since the last questionnaire? |
| 3. | Wenn ja, welche der folgenden Symptome traten auf? | If yes, which of the following symptoms apply? |
|  | Schnupfen | Runny nose |
|  | Halsschmerzen | Sore throat |
|  | Kopfschmerzen | Headache |
|  | Schwindel | Dizziness |
|  | Abgeschlagenheit | Fatigue |
|  | Gliederschmerzen | Aching limbs |
|  | Augenentzündung | Eye inflammation |
|  | Verlust des Geruchssinnes | Loss of sense of smell |
|  | Verlust des Geschmacksinnes | Loss of sense of taste |
|  | Atemnot/ Kurzatmigkeit | Shortness of breath |
|  | Husten | Cough |
|  | Fieber (> 38,5°C) | Fever (> 38.5°C) |
|  | Schüttelfrost | Chills |
|  | Hautausschlag | Skin rash |
|  | Durchfall | Diarrhoea |
|  | Übelkeit | Nausea |
|  | Appetitlosigkeit | Loss of appetite |
|  | Anderes Symptom | Other symptom |
| 4. | Welche anderen Symptome traten auf? | Which other symptoms did the participant have? |

**Questionnaire 3**. Acceptance Questionnaire

| A1 | Datum | Date |
| --- | --- | --- |
| A2 | Jahrgangsstufe | Grade |
| B1 | Nimmt ihr Kind an der STACAMA Studie teil? | Is your child taking part in the STACAMA study? |
| B3 | Waren Sie sich mit ihrem Kind bei der Entscheidung einig? | Did you and your child agree over the decision? |
| B4 | Welche Gründe oder Faktoren haben für Sie und ihr Kind bei der Entscheidung eine Rolle gespielt?  Gründe für Nicht-Teilnahme. | What reasons or factors played a role for you and your child in the decision?  Reasons for not participating. |
| B5 | Welche Gründe oder Faktoren haben für Sie und ihr Kind bei der Entscheidung eine Rolle gespielt?  Gründe für Teilnahme. | What reasons or factors played a role for you and your child in the decision?  Reasons for participating. |
| C1 | Wenn Sie uns noch etwas mitteilen möchten, können Sie das hier  schriftlich tun. Wir freuen uns über jede Anmerkung oder  Rückmeldung. | Further remarks. We appreciate any comments or feedback. |

**Questionnaire 4**. Test Questionnaire.

| A1 | Jahrgangsstufe | | | Grade | | | |
| --- | --- | --- | --- | --- | --- | --- | --- |
| B1 | Haben Sie für die STACAMA-Studie eingewilligt? | | | Did you consent to participation in the STACAMA study? | | | |
| B2 | Nimmt die Klasse / der Jahrgang Ihres Kindes bereits teil? | | | Is your child’s class / grade already participating? | | | |
| B3 | Hat Ihr Kind schon am STACAMA-Testverfahren teilgenommen? | | | Has your child taken part in the STACAMA test procedure yet? | | | |
| C1 | Welches Testverfahren bevorzugen Sie? | das Gurgeltestverfahren (STACAMA): PCR-basiertes Verfahren | | Which test method would you prefer? | | | Gargle test method (STACAMA): PCR-based method |
|  |  | Die Schnelltests in der Schule | |  |  |  | Rapid antigen tests at school |
|  |  | die Schnelltests zuhause (werden zurzeit für das Land Sachsen-Anhalt diskutiert) | |  |  |  | Rapid tests at home (currently under discussion for the state of Saxony-Anhalt) |
| C2 | Aus welche(n) Gründe(n)? |  | | For what reasons? | | | |
| C3 | Welche Art der Probenanalyse würden Sie bevorzugen? | Laborverfahren (PCR-basierte Analyse) | | What type of detection method would you prefer? | | | Laboratory method (PCR-based analysis) |
|  |  | Schnelltestverfahren (Antigenbestimmung) | |  |  |  | Rapid antigen test (antigen determination) |
| C4 | Welche Art der Probenentnahme wäre Ihnen bei freier Auswahl am  liebsten? | Gurgeltest (ausschließlich Laborverfahren) | | What type of sampling method would you prefer if given a free choice? | | Gargle sample (laboratory method only) | |
|  |  |  |  |  |  | Saliva sample | |
|  |  | Spucktest | |  |  |  |  |
|  |  |  |  |  |  | Anterior nasal swab | |
|  |  | Nasenabstrich im vorderen Bereich der Nase | |  |  | Deep nasal swab | |
|  |  | Tiefer Nasenabstrich | |  |  |  |  |
|  |  | Rachenabstrich | |  | | Pharyngeal swab | |
|  |  | Mundrachenabstrich | |  | | Oropharyngeal swab | |
|  |  | Antikörpertest | |  | | Antibody test | |
|  |  | Mundabstrich | |  | | Oral swab | |
| C5 | Aus welche(n) Gründe(n)? | | | For what reasons? | | | |
| C6 | Bevorzugen Sie Tests, die: | vom Kind selbst oder | | Would you prefer tests that are performed by: | The child themselves | | |
|  |  | durch Lehrpersonal | |  | Teaching staff | | |
|  |  | durch medizinisches Personal durchgeführt werden? | |  | | | Medical personnel? |
| C7 | Aus welche(n) Gründe(n)? | | | For what reasons? | | | |
| C8 | Bevorzugen Sie: | | Probenentnahme zu Hause? | Would you prefer sampling: | | | At home? |
|  |  |  | Probenentnahme in der Schule? |  |  |  | At school? |
|  |  |  | Probenentnahme im medizinischen Setting? |  |  |  | In a medical setting? |
| C9 | Aus welche(n) Gründe(n)? | | | For what reasons? | | | |
| D1 | Wenn Sie uns noch etwas mitteilen möchten, können Sie das hier  schriftlich tun. Wir freuen uns über jede Anmerkung oder  Rückmeldung. | | | Further remarks. We appreciate any comments or feedback. | | | |
